# Lack of Clinical Benefit of Thromboprophylaxis in Patients Hospitalized in a Medical Unit Over a 10-year Span

**DOI:** 10.14740/jocmr1712w

**Published:** 2014-02-06

**Authors:** Gabrielle Migner-Laurin, Thomas St-Aubin, Julie Lapointe, Paul Van Nguyen, Robert Wistaff, Mikhael Laskin, Christophe Kolan, Maxime Lamarre-Cliche

**Affiliations:** a Centre Hospitalier de l’Universite de Montreal, Canada; b Faculte de Medecine de l’Universite de Montreal, Canada

**Keywords:** Thromboprophylaxis, Deep vein thrombosis, Pulmonary embolus, Anticoagulation, Hospital medicine

## Abstract

**Background:**

Thromboprophylaxis for hospitalized patients with a high risk of venous thromboembolic events (VTEs) is strongly recommended but is not universally applied on medical units. Outside of randomized trials, there is minimal evidence that the usual medications reduce the incidence of clinically significant VTE.

**Methods:**

We conducted a retrospective cohort study including all patients admitted into a teaching medical unit during years 2001-2002, 2003-2004, 2005-2006, 2007-2008 and 2009-2010. Inclusion criteria for the analysis were having one or more risk factors for a VTE and no contraindication to thromboprophylaxis.

**Results:**

Of 2,369 patients reviewed, 1,302 satisfied the inclusion criteria. Between years 2001-2002 and 2009-2010, the proportion of patients receiving thromboprophylaxis increased from 29.2% to 76.4% (P < 0.0001) and the duration of thromboprophylaxis increased from 63% of hospital stay to 84% (P = 0.004). There was no statistically significant association between the number of risk factors and the rate of thromboprophylaxis. Overall, only 32 patients suffered from a VTE with no decrease in VTE incidence between years 2001-2002 and 2009-2010. A total of 107 patients had a bleeding event, and there was no statistically significant change in the incidence of bleeding during our study period.

**Conclusions:**

In our medical units, we found a statistically significant increase in the use of the thromboprophylaxis practice. However, this was not associated with any statistically significant impact on the VTE incidence. This suggests that patients given thromboprophylaxis could be better selected.

## Introduction

Deep venous thrombosis (DVT) and pulmonary embolism (PE) are well-known complications of immobilized patients on medical units. Most DVTs and PEs are clinically silent and hard to diagnose. The risks factors for having a venous thromboembolic event (VTE) include: age over 75 years, heart failure, a respiratory or inflammatory disease, an active neoplasia, a stroke or a paralysis, a severe infection, a previous VTE and a recent surgery or trauma [1, 2]. About two-thirds of the patients hospitalized with an acute medical condition present with one or more of these risks factors [3-5].

Thromboprophylaxis guidelines have been regularly updated in the past 20 years. In the recommendations published every few years, the American College of Chest Physician (ACCP) recommends thromboprophylaxis for acutely ill medical patients admitted to hospital with high thromboembolic risk. These guidelines have been actively promoted and have been widely implemented. In 2008, the risk factors for DVTs and PEs were congestive heart failure or severe respiratory disease or who are confined to bed and have one or more additional risk factors, including active cancer, previous VTE, sepsis, acute neurologic disease, or inflammatory bowel disease [6]. In 2012, a risk score largely based on the same risk factors help to identify the patients at high risk for VTE.

Randomized-controlled trials and meta-analyses have demonstrated the efficacy of thromboprophylaxis to reduced VTE incidence in patients hospitalized in medical units [7-10]. The pharmacologic methods that have been studied have included unfractionned heparin, low-molecular weight heparins and anti-Xa agents. The results have shown a reduction between 45 and 63% in the incidence of DVT and PE, with a bleeding rate of 0.2 to 1.7%. The majority of DVTs were subclinical. These events were detected because ultrasonograms or venograms were performed in all patients. Patients included in these randomized trials were selected and the follow-up was performed in an ideal research grade environment.

It is unclear if thromboprophylaxis for non-critically ill medical patients has a significant impact on clinically symptomatic VTE outside a research environment. This study’s objective is to measure the evolution and the impact of thromboprophylaxis over a decade in non-selected high-risk patients hospitalized in a single medical unit.

## Method

Retrospective cohort study of all patients admitted to a single Internal Medicine unit at the Hotel-Dieu pavillon of the Centre Hospitalier de l’Universite de Montreal (CHUM-HD) during July 1-June 30th and during the following academic years: 2001-2002, 2003-2004, 2005-2006, 2007-2008 and 2009-2010. The study years were non-consecutive to increase the overall study time span. The study subject had to be considered high risk for VTE according to 2008 ACCP guidelines [6] and could not be anticoagulated at admission.

All files were retrieved from the archives for a revision and only the first hospitalization per patient was used in the analysis. Data collection was performed manually by two research assistants between June and August 2010 and revised over the two following months. The data collected were: patient demographics and hospitalization statistics, presence of VTE risks factors, the nature and duration of thromboprophylaxis, nature/extent of VTE, hemorrhagic complications (severe bleeding defined by a decrease in hemoglobin concentration more than 2 g/L or hemodynamic instability) and occurrence of heparin-induced thrombocytopenia (HIT). The risk factors used for definition of high-risk patients and for estimation of risk severity were found in the 2008 guidelines [6] and are: congestive heart failure, severe respiratory disease, active cancer, previous VTE, sepsis, acute neurologic disease and inflammatory bowel disease. New guidelines were published in 2012 after the end of data collection [11]. These guidelines use the Padua score to define patients at high risk for VTE. This score is based on the 2008 guidelines criteria with the addition of age, body mass index and presence of hormonal treatment. Points are added for each criteria and a patient will be defined as high risk if the sum is equal or greater than 4. The data collected in the present study did not include the last two criteria. Using the available information it was not possible to calculate a precise Padua score but it was possible to identify a subset of patients that had a score at least as high as the high risk threshold. A secondary analysis was performed using this subgroup.

Presence of thromboprophylaxis was defined as the use of any recommended preventive method at any time during the hospital stay of the patient. Absolute thromboprophylaxis duration and proportional thromboprophylaxis duration over the length of in-hospital stay were also calculated for every patient. DVT, PE and VTE (main outcome) rates were calculated as were hemorrhagic or immunologic (HIT) complication rates.

Two main analyses were performed. The first was a descriptive and statistic analysis on measured outcomes over the academic years. The second was a group comparison between patients receiving or not receiving thromboprophylaxis. We used a Fisher exact test for comparison of proportions with Bonferroni correction for tests multiplicity with a 0.05 global signification level. For the comparison of continuous variables, a t test was used. Regression analyses were performed but were limited by the small number of events.

The expected VTE rate was between 5% and 15%. At least 141 high-risk patients per year were needed to enable demonstration of a 10% difference in VTE rate between any two years with a power of 80% and statistical significance of 0.05. The studied internal medicine unit admits between 400 and 500 patients per year and it is estimated that about two-thirds of these patients are at high VTE risk. Taking into account that certain files would not be eligible for the analysis, it was conservatively decided to include every patient admitted during the five study years.

## Results

The entire cohort included 2,369 patients and of those, 1,302 were considered high risk for VTE but not anticoagulated. As shown in Table 1, the baseline characteristics of the entire cohort and the analysis population are similar. Table 2 shows study population demographics divided by year. Over the years, there were less patients admitted with cancer (17% in 2001-2002 to 11% in 2009-2010 (P = 0.0002)). There were no other significant changes over the years for baseline characteristics.

**Table 1:** Baseline Characteristics of the Total Population and the Study Population

	Total population (%)	High risk patients (%)
Number of patients	2369	1302
Male gender	967 (40.8)	567 (43.5)
Female gender	1402 (59.2)	735 (56.5)
Average length of stay (days)	15	18
Age on admission (year)	68	70
Diagnosis on admission		
Non-specified generalized weakness	218 (9.2)	113 (8.7)
Neurologic disease	233 (9.8)	153 (11.8)
Pulmonary disease	65 (2.7)	45 (3.5)
Cardiovascular disease	186 (7.9)	97 (7.5)
Gastrointestinal disease	173 (7.3)	65 (5.0)
Nephro - endo - metab	167 (7.1)	72 (5.5)
Hematologic disease (no cancer)	129 (5.4)	44 (3.4)
Neoplasia	321 (13.6)	286 (21.9)
Rhumatologic and inflammatory	133 (5.6)	40 (3.1)
Infectious disease	363 (15.3)	278 (21.3)
Substance withdrawal or intox	59 (2.5)	33 (2.5)
Dermato - psy - MSK - allergy	184 (7.8)	76 (5.9)
Thromboembolic disease	138 (5.8)	0
Palliative care	222 (9)	196 (15)
Risk factors for VTE		
Average number of risk factors	1.11	1.56
Congestive heart failure	406 (17.1)	305 (23.5)
Severe respiratory condition	570 (24.1)	465 (35.7)
Active cancer or on treatment	435 (18.4)	387 (29.8)
Previous DVT or PE	159 (6.7)	84 (6.5)
Systemic infection or sepsis	479 (20.2)	413 (31.7)
Acute neurologic disease	315 (13.3)	283 (21.8)
Inflammatory bowel disease	32 (1.4)	28 (2.2)
Post-op or trauma < 3 months	79 (3.3)	66 (5.1)

DVT: deep vein thrombosis; MSK: musculosquelettal; PE: Pulmonary embolism; psy: psychiatric; VTE: venous thromboembolic event.

**Table 2:** Baseline Characteristics of the Study Population by Academic Years

	2001-2002	2003-2004	2005-2006	2007-2008	2009-2010
Number of patients	254	326	272	237	214
Male gender	107 (41.9)	141 (43.3)	111 (40.8)	114 (48.1)	95 (44.3)
Female gender	147 (58.1)	185 (56.8)	161 (59.2)	123 (51.9)	118 (55.7)
Average length of stay (days)	19.9	18.8	17.0	16.5	18.2
Age on admission (year)	66.8	69.9	71.7	70.8	71.6
Diagnosis on admission					
Non-specified generalized weakness	25 (9.5)	21 (6.4)	28 (10.3)	20 (8.4)	21 (9.4)
Neurologic disease	28 (11.1)	53 (16.3)	25 (9.2)	25 (10.6)	22 (10.4)
Pulmonary disease	7 (2.8)	5 (1.5)	5 (1.8)	19 (8.0)	9 (4.2)
Cardiovascular disease	21 (8.3)	19 (5.8)	24 (8.8)	17 (7.2)	16 (7.6)
Gastrointestinal disease	7 (2.8)	15 (4.6)	20 (7.4)	13 (5.5)	10 (4.7)
Nephro - endo - metab	14 (5.5)	15 (4.6)	25 (9.2)	12 (5.1)	6 (2.8)
Hematologic disease (no cancer)	11 (4.4)	11 (3.4)	9 (3.3)	7 (2.9)	6 (2.8)
Neoplasia	72 (28.5)	77 (23.6)	37 (13.6)	44 (18.6)	35 (16.5)
Rhumatologic and inflammatory	2 (0.8)	14 (4.3)	8 (2.9)	9 (3.8)	7 (3.3)
Infectious disease	53 (20.9)	66 (20.2)	46 (16.9)	48 (20.3)	64 (30.2)
Substance withdrawal or intox	7 (2.8)	6 (1.8)	6 (2.2)	6 (2.5)	8 (3.8)
Dermato - psy - MSK - allergy	7 (2.77)	24 (7.36)	19 (6.98)	17 (7.17)	9 (4.24)
Thromboembolic disease	0	0	0	0	0
Palliative care	52 (20.6)	34 (10.4)	52 (19.1)	25 (10.6)	32 (15.1)
Risk factors for VTE					
Congestive heart failure	55 (21.8)	60 (18.4)	99 (36.4)	45 (19.0)	46 (21.7)
Severe respiratory condition	89 (35.2)	102 (31.3)	106 (39.0)	91 (38.4)	76 (35.9)
Active cancer or on treatment	84 (32.8)	108 (33.1)	77 (28.3)	66 (27.9)	53 (25.0)
Previous DVT or PE	18 (7.1)	21 (6.4)	19 (7.0)	13 (5.5)	14 (6.1)
Systemic infection or sepsis	81 (32.0)	104 (31.9)	64 (23.5)	67 (28.3)	96 (45.3)
Acute neurologic disease	44 (17.4)	90 (27.6)	51 (18.8)	48 (20.3)	50 (23.6)
Inflammatory bowel disease	3 (1.2)	5 (1.5)	3 (1.1)	11 (4.6)	6 (2.8)
Post-op or trauma < 3 months	11 (4.4)	24 (7.4)	10 (3.7)	9 (3.8)	8 (3.8)

Percentage in parentheses. DVT: deep vein thrombosis; MSK: musculosquelettal; PE: pulmonary embolism; psy: psychiatric; VTE: venous thromboembolic event.

**Figure 1:**
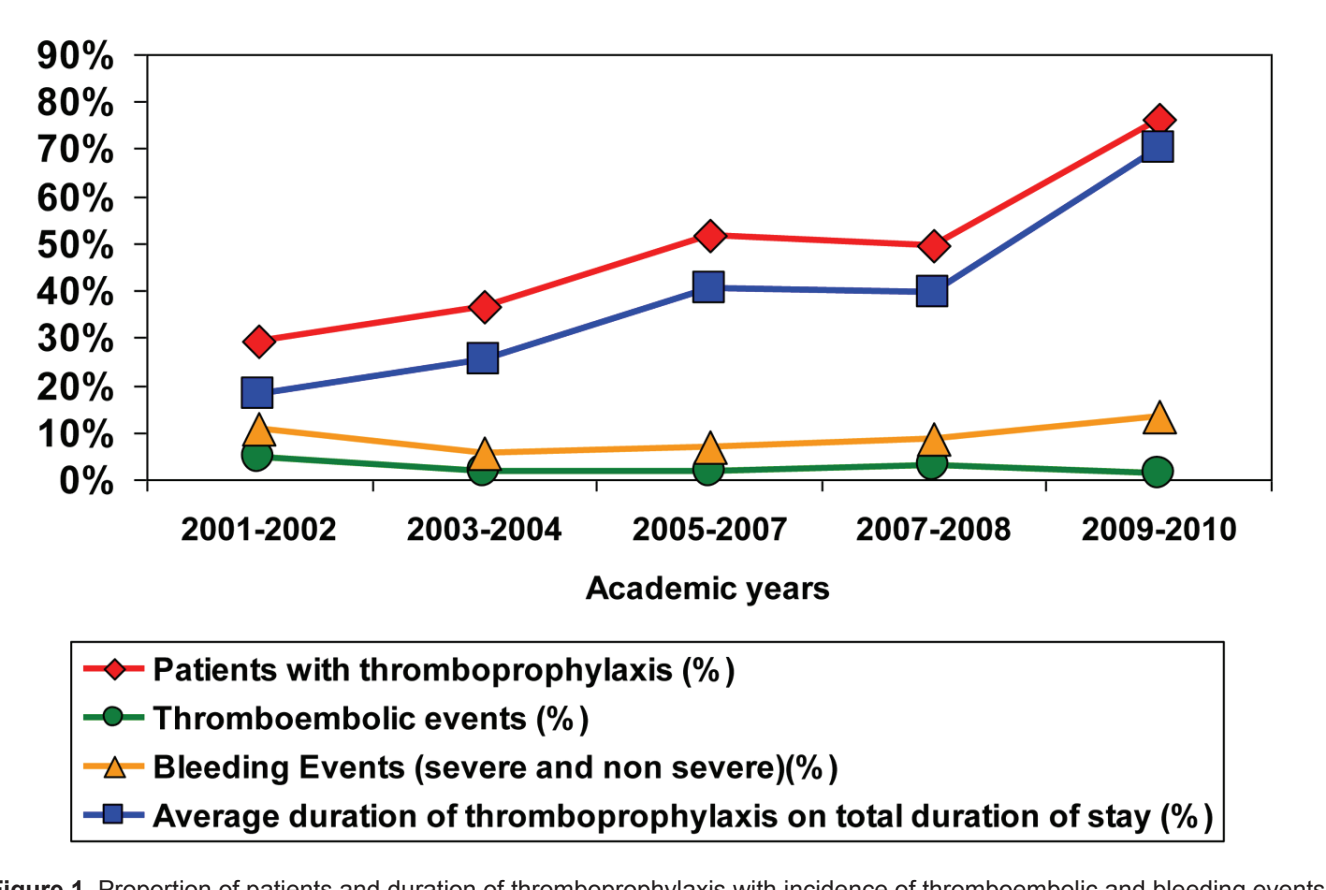
Proportion of patients and duration of thromboprophylaxis with incidence of thromboembolic and bleeding events.

Figure 1 shows a significant increase in thromboprophylaxis rates over the study period (29.2% in 2001-2002 to 76.4% in 2009-2010 (P < 0.0001)). There was an increase in the proportional duration of thromboprophylaxis on total length of stay from 18% in 2001-2002 to 70% in 2009-2010. Proportion of patients prescribed LMWH increased continuously from 2% to 44%, whereas UFH decreased over the studied years (P < 0.0001).

There were 32 in-hospital symptomatic and confirmed VTE diagnoses and 111 hemorrhagic complications (severe and non-severe combined). Throughout the studied years, the percentage of patients with a VTE ranged between 1.4 and 4.7%. No significant tendency could be shown for VTE (P = 0.124) or DVTs (P = 0.426), or PEs (P = 0.115) or the incidence of bleeding (P = 0.151). Only one patient had an HIT diagnosis confirmed in 2009.

Table 3 illustrates that patients with thromboprophylaxis had more risk factors for VTE and appeared to have a greater disease burden. There was a greater incidence of PE (P = 0.03) and a non-statistically significant tendency towards a greater VTE incidence (P = 0.051) in patients receiving thromboprophylaxis. The incidence of bleeding was similar between the two groups.

**Table 3:** Risk Factors, VTE and Complications Rate Between the Patients With or Without Thromboprophylaxis

	Patients with thromboprophylaxis	Patients without thromboprophylaxis
Number of patients	615	687
Palliative care	94 (15.3)	101 (14.7)
Risk factors for VTE		
Congestive heart failure	162 (26.4)	143 (20.9)
Severe respiratory condition	225 (36.6)	239 (34.8)
Active cancer or on treatment	171 (27.8)	216 (31.5)
Previous DVT or PE	42 (8.8)	42 (6.1)
Systemic infection or sepsis	214 (34.9)	198 (28.9)
Acute neurologic disease	150 (24.4)	133 (19.4)
Inflammatory bowel disease	13 (2.1)	15 (2.2)
Post-op or trauma < 3 months	54 (8.8)	12 (1.8)
Thromboembolic events		
Total thromboembolic disease	21 (3.4)	11 (1.6)
Proximal DVT	5 (0.8)	4 (0.6)
Distal DVT	7 (1.1)	6 (0.9)
Pulmonary embolism	10 (1.6)	2 (0.3)
Fatal pulmonary embolism	3 (0.5)	1 (0.1)
Complications		
Total bleeding events	50 (8.1)	61 (8.9)
Non-severe bleeding	21 (3.4)	35 (5.1)
Severe bleeding	29 (4.7)	26 (3.8)
HIT	1 (0.2)	0

Percentage in parentheses. DVT: deep vein thrombosis; HIT: heparin-induced thrombocytopenia; PE: pulmonary embolism; VTE: venous thromboembolic event.

There was no statistical association between the pharmacologic method used (standard or low molecular weight heparin) and the rate of VTE or bleeding. Patients with one, two and three or more risk factors had a VTE rate of 2.2%, 2.6% and 4.1% respectively.

Further analysis of the subgroup of 1,018 patients with a Padua score of 4 did not yield significantly different results. However, increased Padua score was associated with increased risk of thromboembolic events (22% relative increase with each added point in the score) and likelihood of receiving thromboprophylaxis (26% relative increase with each added point in the score).

## Discussion

This study shows an increase in the rate and duration of thromboprophylaxis over a decade in a medical unit. Notably, this change in practice was not associated with a statistically significant decrease in symptomatic VTE. A link between thromboembolic risk and VTE may exist but could not be demonstrated in our study due to the low number of events. This study offers a useful longitudinal perspective of the population in a standard medical unit. Certain limitations are inherent to the retrospective nature of the study design but our hope is that the results reflect in-hospital practice as compared to the clinical research setting.

The increase in thromboprophylaxis over the past decade was predictable given consecutive guidelines promoting VTE prevention in high risk medical patients. Furthermore, an admission protocol has been developed in 2009 in the study site. It includes a section on thromboprophylaxis, which serves as a trigger for the clinician to prescribe it depending unless there are contra-indications. These practice changes presumably reflect widespread acceptance that most hospitalized medical patients are both at risk for VTE and will benefit from preventive measures.

The results from this cohort study were surprising. The incidence of clinically significant VTE was very low compared to published clinical trials. In the PREVENT trial, total VTE rates were 3.1% [9]. This number increased to 8.1% in the ARTEMIS trial [8] and to 11.8 % in MEDENOX trial [10]. However, our results were a valid local representation of five full years of a medical hospital unit over a decade. Though thromboprophylaxis may offer subclinical benefits, it did not provide with clinically significant absolute reduction in VTE events. A greater number of patients could have helped identify a statistically significant effect of thromboprophylaxis but the clinical relevance of a small absolute benefit may still be unclear. A clearer association might be demonstrable with patients at higher VTE risk.

Total bleeding events in the two groups was more prevalent than the total VTE over the 5 years of the study which may seem unusual. In this retrospective cohort study, identification of bleeding may have been influenced by the limitations of study design. Our study patients may have had comorbidities which could have translated into a greater propensity to bleed.

The American College of Physicians (ACP) has recently modified their guidelines following a systematic review that demonstrated no improvement upon mortality combined with an increase in bleeding [12, 13]. It states that the thromboembolic and the bleeding risks have to be carefully balanced, rather than prescribing thromboprophylaxis routinely to all patients with one or more thrombotic risk factor. The results from our study are in agreement with this change in recommendations.

### Conclusion

A significative increase in the use thromboprophylaxis over the last 10 years had no discernable impact on the incidence of clinical VTE in hospitalized medical patients with risk factors. Better selection criteria would help identify patients who could gain most and be harmed least.
